# A Compatible Control Algorithm for Greenhouse Environment Control Based on MOCC Strategy

**DOI:** 10.3390/s110303281

**Published:** 2011-03-18

**Authors:** Haigen Hu, Lihong Xu, Bingkun Zhu, Ruihua Wei

**Affiliations:** 1 Department of Control Science and Engineering, Tongji University, Shanghai 200092, China; E-Mails: zhu1981 2001@yahoo.com.cn (B.Z.); laurrywei@sina.com (R.W.); 2 School of Information Engineering, Zhejiang Agriculture & Forestry University, Lin’an City 311300, Zhejiang Province, China; E-Mail: hnhhg@163.com

**Keywords:** multi-objective compatible control (MOCC), greenhouse environment control, feedback control, multi-objective evolutionary algorithms (MOEAs), greenhouse climate model

## Abstract

Conventional methods used for solving greenhouse environment multi-objective conflict control problems lay excessive emphasis on control performance and have inadequate consideration for both energy consumption and special requirements for plant growth. The resulting solution will cause higher energy cost. However, during the long period of work and practice, we find that it may be more reasonable to adopt interval or region control objectives instead of point control objectives. In this paper, we propose a modified compatible control algorithm, and employ Multi-Objective Compatible Control (MOCC) strategy and an extant greenhouse model to achieve greenhouse climate control based on feedback control architecture. A series of simulation experiments through various comparative studies are presented to validate the feasibility of the proposed algorithm. The results are encouraging and suggest the energy-saving application to real-world engineering problems in greenhouse production. It may be valuable and helpful to formulate environmental control strategies, and to achieve high control precision and low energy cost for real-world engineering application in greenhouse production. Moreover, the proposed approach has also potential to be useful for other practical control optimization problems with the features like the greenhouse environment control system.

## 1. Introduction

The adjustment of greenhouse environment has a great influence on plant growth, production yield, quality and energy consumption. In order to achieve high yield at low expense, good quality and low environmental load, several parameters such us temperature, air humidity and CO_2_ concentration must be controlled optimally through heating, fogging, ventilation and CO_2_ injection. Moreover, the goals of high precision and low energy consumption are always desired simultaneously. On one hand, high precision in a control system generally means high energy consumption. However, on the other hand, we are simultaneously required to minimize energy consumption to reduce the cost. The two desired goals are always in conflict with each other. Therefore, a class of multi-objective conflict control problems must be solved to achieve the above goals.

In recent years, multi-objective conflict control problems have attracted great interest and have been extensively studied due to their application in many fields. There are two important conventional methods to solve such problems. One is the trade-off weight method [1], which aggregates the multiple objectives into one overall objective function by adding a set of trade-off weights [1–3]. It is very simple and easy to deal with, but the control result will heavily depend on the selection of trade-off weights, and the result may not be satisfactory if unsuitable weights were assigned. Moreover, it is very difficult for users and engineers to select suitable weights in real-world engineering applications, and it is not also appropriate for non-convex problems to handle. Another highly important method for multi-objective control is the constraints method, which optimizes the most important control objective and translates the others into system constraints [4–6]. The advantage of this approach is to satisfy all controlled objectives through constraints. However, it is very difficult for users or control engineers to determine suitably the constraint bounds in a practical problem. Bounds that are too tight may bar the existence of a feasible solution for the optimization problem, while unduly loose bounds may make the optimization problem lose practical significance.

In the past ten years, we have studied the greenhouse environment control problem and have gained a considerable understanding in controlling the properties of greenhouse environment. When the conventional methods were adopted, we could maintain the temperature and humidity at a very precise point, but the high energy consumption and expensive cost of this strategy would make the greenhouse unprofitable, which implies that this control strategy would not be chosen by any users. Another main attention is that the conventional methods sometimes cannot ensure the existence of a feasible controller in advance. Considering that the traditional multi-objective control methods are unsatisfactory and inappropriate for greenhouse environment control problems, we attempt to adopt a multi-objective coordinated control system in the greenhouse. When these objectives conflict with each other, it is impractical to fix all the objectives at some given optimal points. Thus, we are willing to back off on our desire that all controlled objectives be precisely at their optimal values, relaxing these point controlled objectives to some suboptimal intervals or regions, more generally, we call them compatible objective regions. This method is called multi-objective compatible control (MOCC).

This type of problem has two distinctive characteristics: (1) There exist multiple conflicting control optimization objectives. (2) These controlled objectives can be allowed to settle for suboptimal solutions owing to the trade-off between multiple competitive specifications. Such problems are also widely found in industrial control. For example, the control of urban traffic flow is also a typical complex multi-objective control problem, characterized by the conflict between the main roads and support roads in the saturation state. Intensive research has focused on this problem to improve traffic management [7]. MOCC has also been applied to this traffic flow control problem [8], including a Ph.D. dissertation written on the subject [9]. In this work, a modified compatible control algorithm by adopting interval or region control objectives instead of point control objectives is proposed to solve such multi-objective conflict control problems, and a greenhouse climate control model with feedback is adopted to test the performance of the proposed algorithm.

The paper is organized as follows: Section 2 describes the MOCC framework used in this work. In section 3, the greenhouse climate model and its derivation are given. In Section 4, the control model is described and a modified MOCC algorithm is proposed. The simulation experiments and operation parameters are presented in Section 5. In Section 6, analysis and discussion on experiment results are presented. Section 7 gives concluding remarks and some directions for further research.

## 2. Multi-Objective Compatible Control Framework

**Figure 1.**
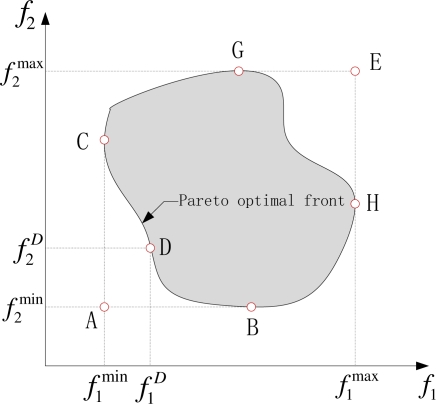
Two-objective conflict control problem.

Conventional multi-objective control methods always select the optimal value of each performance as the point control objective and minimize the deviation of each performance. However, it is very difficult to achieve the multiple objectives’ precise points simultaneously due to the conflicts between them. For example, given a two-objective conflict control problem in Figure 1, we always desire obtaining the minimal point *A* of the two objectives *f*_1_ and *f*_2_ simultaneously. In reality, the feasible region with control solutions existing is the shaded area. This happens to other multi-objective conflict control problems, so it is utopian and impractical for multi-objective conflict control problems to adopt the method of the precise point control in practical applications.

Considering the above problems and based on the successful application in greenhouse environment control, Xu *et al.* proposed a two-layer compatible control framework [10] and a modified Multi-objective Compatible Control (MOCC) algorithm [11] by using some suboptimal interval or region control objectives (*i.e*., compatible objective regions) instead of the optimal point control objectives, namely enlarging the point objectives to intervals or regions to ensure the existence of the controller. For instance, the ranges of $\left[f_{1}^{\min},\;f_{1}^{\max}\right]$ and $\left[f_{2}^{\min},\;f_{2}^{\max}\right]$ are generally selected as their respective compatible objective regions, and any point *D* of the Pareto optimal front *BCD* is the desired solution by adopting compatible optimization to achieve (shown in Figure 1). Based on the algorithm, some research and successful applications have been performed in the literatures [7,9,12–14].

**Figure 2.**
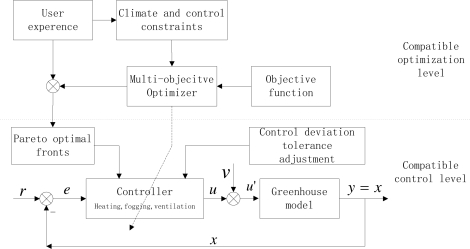
Modified two-layer compatible control framework.

On the basis of the previous work and some successful experiences, a modified compatible control framework is proposed in this work (shown in Figure 2). From this framework, we also adopt two hierarchical levels, referred as the compatible optimization level and compatible control level. In the compatible optimization level, climate conditions and control constraints are determined according to the special requirements for plant growth and user’s experiences of greenhouse production. Then, we can achieve the Pareto optimal fronts by using MOEAs. But in the compatible control level, the corresponding variables (such as heating, fogging and ventilation) of the obtained Pareto optimal fronts are selected as the input vector *u* of the control system. Then, we can implement the corresponding optimal control by adopting feedback control architecture and adjusting the control deviation tolerance.

## 3. Greenhouse Environment Model

The greenhouse environment is a complex dynamical system, and temperature and humidity are highly coupled through nonlinear thermodynamic laws. Over the past decades, people have gained a considerable understanding of greenhouse climate dynamics, and many dynamic climate models of a greenhouse have been proposed [15–19]. Most of the studies on analysis and control of the environment inside greenhouses have been based on the model of the following state space form:

$$\dot{x}=f\left(t,\;x,\;u,\;v\right)$$

where *x* are states variables like indoor temperature, humidity and carbon dioxide concentration, *u* are control inputs like energy input by the heating system, fogging systems, ventilation system and *CO*_2_ supply flux, *v* are external disturbances like solar radiation, outdoor temperature, humidity and wind speed, *t* denotes time, and *f* is a nonlinear function.

For application of optimal control, an accurate model of the controlled processes is necessary. A simple greenhouse heating/cooling/ventilating model can be obtained from many extant literatures in this work. The state equations have been formed based on the laws of conservation of enthalpy and matter, and the dynamic behavior of the states is described by using the following differential equations:

(1) $$\frac{{\mathrm{dT}}_{in}\;\left(t\right)}{\mathrm{dt}}=\frac{1}{\rho C_{p}V_{T}}\left[Q_{\mathrm{heater}}\;\left(t\right)+S_{i}\;\left(t\right)-\lambda Q_{\mathrm{fog}}\;\left(t\right)\right]-(\frac{V_{R}\;\left(t\right)}{V_{T}}+\frac{\mathrm{UA}}{\rho C_{p}V_{T}})\left[T_{\mathrm{in}}\;\left(t\right)-T_{\mathrm{out}}\;\left(t\right)\right]$$

(2) $$\frac{{\mathrm{dw}}_{\mathrm{in}}\;\left(t\right)}{\mathrm{dt}}=\frac{1}{V_{H}}Q_{\mathrm{fog}}\;\left(t\right)+\frac{1}{V_{H}}\left[E\left(S_{i}\;\left(t\right),w_{\mathrm{in}}\;\left(t\right)\right)\right]-\frac{V_{R}\;\left(t\right)}{V_{H}}\left[w_{\mathrm{in}}\;\left(t\right)-w_{\mathrm{out}}\;\left(t\right)\right]$$

(3) $$E\left(S_{i}\;\left(t\right),w_{\mathrm{in}}\;\left(t\right)\right)=\alpha \frac{S_{i}\;\left(t\right)}{\lambda }-\beta_{T}w_{\mathrm{in}}\;\left(t\right)$$

where

*T_in_/T_out_* is the indoor/outdoor air temperature (°C),
*w_in_/w_out_* is the indoor/outdoor relative humidity (%),
*UA* is the heat transfer coefficient of enclosure (*W K*^−1^),
*V* is the geometric volume of the greenhouse (*m*^3^),
*ρ* is the air density (1.2*kgm*^−3^),
*C_p_* is the specific heat of air (1006*Jkg*^−1^*K*^−1^),
*Q_heater_* is the heat provided by the greenhouse heater (*W*),
*Q_fog_* is the water capacity of the fog system (*gH*_2_*Os*^−1^),
*S_i_* is the intercepted solar radiant energy (*W*),
λ is the latent heat of vaporization (2257*Jg*^−1^),
*V_R_* is the ventilation rate (*m*^3^*s*^−1^),
*E*(*S_i_*, *w_in_*) is the evapotranspiration rate of the plants (*gH*_2_*Os*^−1^), which is affected by the given solar radiation,
*α* and *β_T_* are scaling parameters, which are considered as constant over a short period due to their relatively low-frequency variation,
*V_T_* and *V_H_* are the temperature and humidity of the actively mixing air volumes, respectively. Generally speaking, *V_T_* and *V_H_* are as small as 60%–70% of the geometric volume *V* of the greenhouse (see [20] for details).

The central state variables are air temperature and relative humidity of inside greenhouse, and control inputs come from heating, ventilation and fogging. Disturbances to a greenhouse occur primarily from solar radiation, outside temperature and humidity. Generally, considering that the conditions of operating the ventilation/cooling are rather dominated by solar radiation alone (*i.e*., β*_T_* = 0), the term β*_T_w_in_*(*t*) in Equation (3) can be neglected. Supposing that *C*_0_ = *ρC_p_V_T_* and *α*′ = *α*(λ*V_H_*)^−1^, and normalizing the control variables through the following equations:

$$\begin{array}{c} Q_{\mathrm{heater},\%}=Q_{\mathrm{heater}}/Q_{\mathrm{heater}}^{\max},\\ V_{R,\%}=V_{R}/V_{R}^{\max},\\ Q_{\%,\mathrm{fog}}=Q_{\mathrm{fog}}/Q_{\mathrm{fog}}^{\max},\\ \lambda \prime =\lambda Q_{\mathrm{fog}}^{\max}, \end{array}$$

and

$$V\prime =V_{H}/Q_{\mathrm{fog}}^{\max}.$$

Then, the system equations of Equations (1) and (2) are modified as follows:

(4) $$\frac{{\mathrm{dT}}_{\mathrm{in}}\;\left(t\right)}{\mathrm{dt}}=\frac{1}{C_{0}}\left[Q_{\mathrm{heater}}^{\max}Q_{\mathrm{heater},\%}\;\left(t\right)+S_{i}\;\left(t\right)-\lambda \prime Q_{\%,\mathrm{fog}}\;\left(t\right)\right]-(\frac{V_{R,\%}\;\left(t\right)}{t_{v}}+\frac{\mathrm{UA}}{C_{0}})\left[T_{\mathrm{in}}\;\left(t\right)-T_{\mathrm{out}}\;\left(t\right)\right]$$

(5) $$\frac{{\mathrm{dw}}_{\mathrm{in}}\;\left(t\right)}{\mathrm{dt}}=\frac{Q_{\%,\mathrm{fog}}\;\left(t\right)}{V\prime }+\alpha \prime S_{i}\;\left(t\right)-\frac{V_{R,\%}\;\left(t\right)}{t_{v}}\left[w_{\mathrm{in}}\;\left(t\right)-w_{\mathrm{out}}\;\left(t\right)\right]$$

Here parameter *t_v_* represents the time needed for one air change the sampling period.

## 4. Multi-Objective Compatible Control of Greenhouse Environment

### 4.1. Greenhouse Environment Control Problem

The greenhouse climate control problem is to create a favorable environment for the crop in order to reach predetermined results for high yield, high quality and low costs. However, it is a very difficult control problem to implement in practice due to the complexity of the greenhouse environment. For example, the state variables are highly correlated and coupled, and the greenhouse climate is largely perturbed by the outside weather (wind velocity, outside temperature and humidity, *etc*.) and also by many other practical constraints (actuators, moistening cycle, etc.). In recent years, the control design of the climatic conditions in greenhouses is receiving increased attention from many research communities, and many related strategies and control techniques have been proposed, such as various types predictive control [21–23], adaptive control [24,25], nonlinear feedback control [26], fuzzy control [27–29], robust control [30,31] and optimal control [32–34]. These studies are very important to real-world engineering application in greenhouse production.

However, most previous studies lay excessive emphasis on control performance and have inadequate consideration for both energy consumption and special requirements for plant growth. In fact, most plants (like humans) normally thrive within a comfort zone of humidity and temperature. Physiological studies have also shown that for many crops it is sufficient to maintain an average temperature and humidity in a greenhouse over a period [35,36]. So, the main aim of the climate control problem is to maintain the variables (*i.e.*, temperature and humidity) defining the inside greenhouse environment within suitable ranges, not a precision setpoint. Moreover, control performance and energy consumption are two conflicting objectives with each other, and high control precision generally means high energy load. Thus, in order to achieve high yield and as low cost as possible, we must only maintain the temperature and humidity within an acceptable region which is suitable for the plant growth. For example, we regulate the temperature objective to be in the interval 22 °C–28 °C instead of average temperature 25 °C and the humidity objective to 60%–80% instead of average relative humidity 70%.

### 4.2. Control Model

In this section, the multi-objective compatible control strategy presented in Section 2 is applied to the problem of greenhouse heating, ventilation and moisturizing. The greenhouse model, Equations (4) and (5), cannot be put into the rather familiar form of an affine analytic nonlinear system. From the presented compatible control level in Figure 2, the control system adopts a simple feedback control architecture, where desirable target conditions (reference points), control inputs, state variables and disturbance variables can be written the following case respectively

$$\begin{array}{c} r={\left(\begin{array}{cc} r_{1} & r_{2} \end{array}\right)}^{T}={\left(\begin{array}{cc} T_{\mathrm{in}}^{r} & w_{\mathrm{in}}^{r} \end{array}\right)}^{T}\\ u={\left(\begin{array}{ccc} u_{1} & u_{2} & u_{3} \end{array}\right)}^{T}={\left(\begin{array}{ccc} Q_{\mathrm{heater},\%} & Q_{\%,\mathrm{fog}} & V_{R,\%} \end{array}\right)}^{T}\\ x={\left(\begin{array}{cc} x_{1} & x_{2} \end{array}\right)}^{T}={\left(\begin{array}{cc} T_{\mathrm{in}} & w_{\mathrm{in}} \end{array}\right)}^{T}\\ v={\left(\begin{array}{ccc} v_{1} & v_{2} & v_{3} \end{array}\right)}^{T}={\left(\begin{array}{ccc} T_{\mathrm{out}} & w_{\mathrm{out}} & S_{i} \end{array}\right)}^{T} \end{array}$$

supposing the following analytic functions

$$A\left(x,v\right)=\left(\begin{array}{c} \frac{S_{i}-\mathrm{UA}\left(T_{\mathrm{in}}\;(t)-T_{\mathrm{out}}\;(t)\right)}{C_{0}}\\ \lambda \prime S_{i} \end{array}\right)$$

and

$$B\left(x,v\right)=\left(\begin{array}{ccc} \frac{1}{C_{0}} & -\frac{\lambda \prime }{C_{0}} & -\frac{T_{\mathrm{in}}\;\left(t\right)-T_{\mathrm{out}}\;\left(t\right)}{t_{v}}\\ 0 & \frac{1}{V\prime } & -\frac{w_{\mathrm{in}}\;\left(t\right)-w_{\mathrm{out}}\;\left(t\right)}{t_{v}} \end{array}\right).$$

Then, we can get the nonlinear continuous-time system given by the following state equations from the above control model:

(6) $$\dot{x}=A\left(x,v\right)+B\left(x,v\right)u\;\;y=x$$

### 4.3. Multi-Objective Compatible Control Algorithm

#### 4.3.1. Overall Procedure

**Figure 3.**
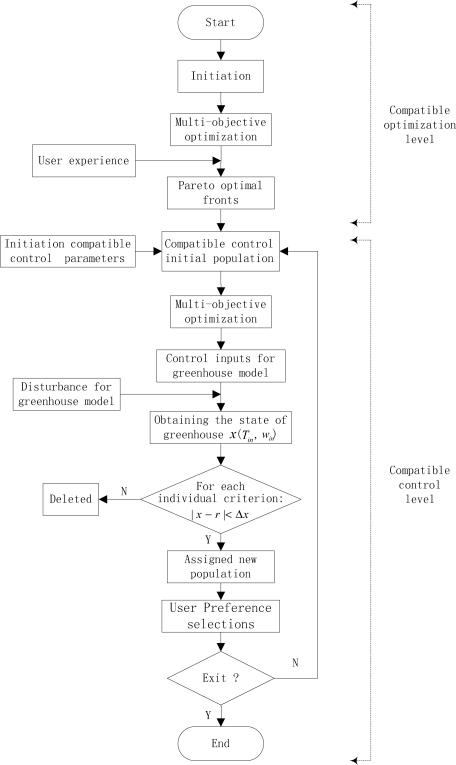
Overall procedure of the MOCC for greenhouse environment.

According to the above description, the overall procedure of the compatible control strategy is shown in Figure 3. Two levels both have a multi-objective optimization stage. The former focus on searching Pareto optimal fronts and achieving the initial population required of the latter. In contrast, the latter emphasizes searching the optimal control inputs to maintain them within compatible objective regions, with as low energy consumption and control error as possible, according to the feedback and the control deviation tolerance Δ*x*. This is the main difference between them.

#### 4.3.2. Multi-Objective Optimization

In order to achieve less energy consumption and higher control precision, and considering that the energy functions in the control theory are generally adopted a quadratic form, we construct the following error objective function:

(7) $$f_{\mathrm{error}}\;\left(t\right)=\sum {\left(\frac{x-r}{r}\right)}^{2}=\sum_{i=1}^{n}{\left(\frac{x_{i}-r_{i}}{r_{i}}\right)}^{2}\;n=1,2$$

and energy consumption objective function:

(8) $$f_{\mathrm{energy}}\;\left(t\right)=\sum \omega {\left({\mathrm{uu}}^{\max}\right)}^{2}=\sum_{i=1}^{m}\omega_{i}{\left(u_{i}u_{i}^{\max}\right)}^{2}$$

$$\begin{array}{c} \sum_{i=1}^{m}\omega_{i}=1\;m=1,2,3\\ 0\leq u_{i}\leq 1,\\ 0\leq \omega_{i}\leq 1. \end{array}$$

where *u^max^* are the respective max energy consumption of control inputs given as follows:

$$u^{\max}={\left(\begin{array}{ccc} Q_{\mathrm{heater}}^{\max} & Q_{\mathrm{fog}}^{\max} & Q_{R}^{\max} \end{array}\right)}^{T}$$

and *ω* is the weight, selected according to the practical experience and the respective equipment power. We consider that the heating system and spraying system are high-energy-consumption equipments, here *u_i_* = 0.75,0.2, 0.05, respectively. Therefore, to achieve the desire goals, the above two objective functions should be minimized simultaneously.

**Figure 4.**
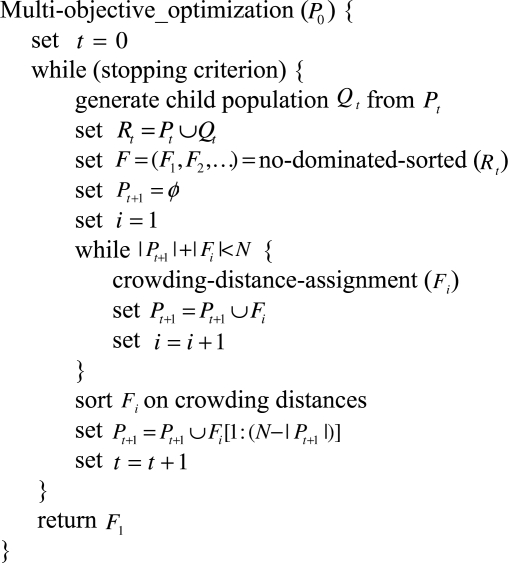
Multi-objective optimization algorithm.

For multiple-objective optimization problems, there has been an increasing interest in applying evolutionary algorithms due to the relevance for real world applications over last twenty years. Multi-Objective Evolutionary algorithms (MOEAs), as a class of effective optimization methods, are used to obtain Pareto solutions for multiple conflicting objectives. Many good methods, such as NSGA-II [37], SPEA2 [38] and PAES [39], appear to be very promising ways to approximate Pareto fronts. Because of its good distribution and convergence, we attempt to employ a modified NSGA-II for compatible optimization to obtain the minimum control error and energy consumption in this work. Compared with the original NSGA-II, the algorithm subtracts the population initialization stage and maintain the main body of NSGA-II(see Figure 4).

#### 4.3.3. Compatible Optimization Level

**Figure 5.**
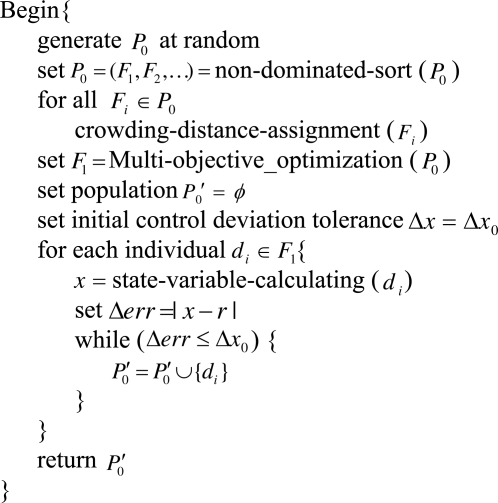
Compatible optimization level algorithm.

In this section, we describe the compatible optimization level, in which the state variables (*i.e.*, indoor temperature and humidity) are incorporated into the algorithm. Firstly, some parameters are initialized and the initial population is generated by some traditional methods. Then the multi-objective optimizer is called for Pareto optimal front search, and finally the obtained population *P′*_0_ with solutions of the front *F*_1_, within the initial control deviation tolerance Δ*x*_0_, is selected as the initial population of the compatible control level. The pseudo code of the compatible optimization level is shown in Figure 5.

#### 4.3.4. Compatible Control Level

**Figure 6.**
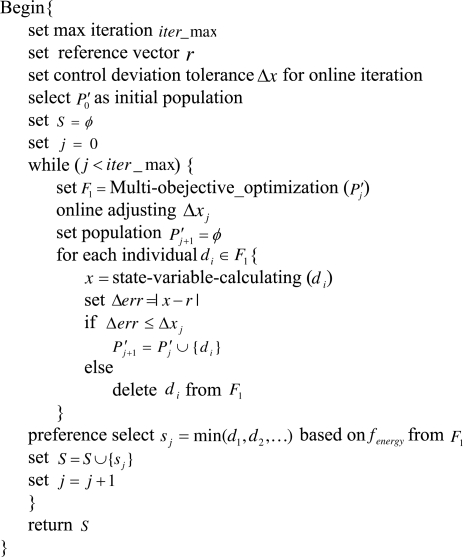
Compatible control level algorithm.

From the above compatible optimization level algorithm, we can obtain the required initial population *P′*_0_ within control deviation tolerance. Based on the objectives of high control precision and as low energy consumption as possible, we can achieve a series of Pareto optimal solution sets of control inputs by calling the Multi-objective optimizer. Then, the indoor temperature (*T_in_*) and humidity (*w_in_*) are regulated within the compatible objective regions by using the online iterative and adjustment method. Finally, we select the solution with the lowest energy consumption from the obtained front *F*_1_ at each iteration. The pseudo code of this approach is given in Figure 6.

## 5. Simulation Experiments

A series of simulation experiments using the proposed compatible control algorithm based on the greenhouse climate model are presented. The operators and parameters in Table 1 are used. Each individual in the evolutionary algorithm represents the control input vector *u* (*i.e.*, heating (*Q*_%,*heater*_(*t*)), ventilation (*V*_*R*,%_(*t*)) and fogging (*Q*_%,*fog*_(*t*))). Let the area of greenhouse be 1000 *m*^2^ and the height be 4 *m*. The greenhouse is equipped by a shading screen, which reduces the transmitted solar radiant energy by 50%. The maximum water capacity of the fog system is 26 *gmin*^−1^*m*^−3^. Maximum ventilation rate corresponds to 20 changes of the greenhouse air per hour. The maximum heating energy is 150 *W m*^−2^. The parameters associated with the greenhouse model had been achieved through the preliminary identification tests in [26]. They are shown in Table 2. In this table, the parameters are expressed per square meter (*m*^2^) of greenhouse area.

In order to effectively validate the performance and to test the robustness of our method, we supposed the disturbance of greenhouse environment as follows:

$$v=\left(\begin{array}{c} v_{1}\\ v_{2}\\ v_{3} \end{array}\right)=\left(\begin{array}{c} T_{\mathrm{out}}\\ w_{\mathrm{out}}\\ S_{i} \end{array}\right)=\left(\begin{array}{c} 2*\mathrm{randf}()\\ 25+15*\mathrm{randf}()\\ 5+5*\mathrm{randf}() \end{array}\right)$$

where *randf*() is a general random function correlated with system time to keep different value when called each time, and its value range is [0,1] with the precision of 0.001.

Based on the experiences in greenhouse production, we generally select the average values of the suitable climate for plant growth as the reference vector *r*. For the convenience of processing, we select the midpoint values of the upper and lower limits of compatible objective regions as the reference vector *r* in this work. Besides, we select the worst acceptable state vector values, which only ensure the plants to survive, but not to flourish, as the initial compatible objective regions. The climate conditions and related parameters are given in Table 3. Hence, we can obtain the following relational functions between the reference vector *r*, control deviation tolerance Δ*x* and the limits of compatible objective regions.

$$r=\frac{x_{\min}+x_{\max}}{2}$$

$$\Delta x=\frac{x_{\max}-x_{\min}}{2}$$

Then, we can obtain the reference vector *r* = (25 65)*^T^* and the initial control deviation tolerance Δ*x*_0_ = (10 20)*^T^* .

## 6. Results and Discussion

This section outlines representative control results by applying the proposed algorithms.

**Figure 7.**
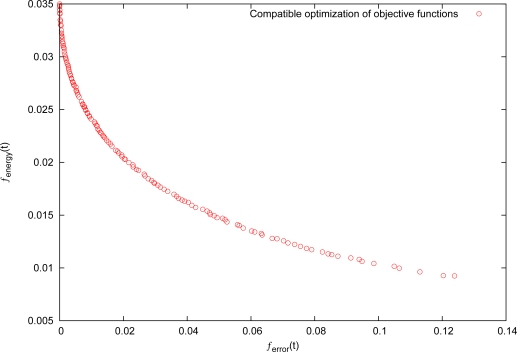
Objective conflict between energy consumption and control precisions.

Figure 7 shows the result obtained by the use of the compatible optimization level. It is clearly seen that the energy consumption is always in conflict with the control precisions. To achieve different convergence rate, we adopt various adjustment methods for control deviation tolerance Δ*x* at each iterative, and discuss the following various cases.

### 6.1. Case 1: Arithmetic Sequence Adjustment

Suppose the control deviation tolerance Δ*x* (*i.e.*, Δ*T* and Δ*w*) is given by the following arithmetic sequence adjustment at each iteration:

$$\begin{array}{c} \Delta x_{i+1}=\Delta x_{i}-\Delta \\ i=0,1,2,\ldots ,\mathrm{iter}\_\max-1. \end{array}$$

where Δ is a constant vector, and we select Δ = (0.32 0.63)^*T*^ in this work.

**Figure 8.**
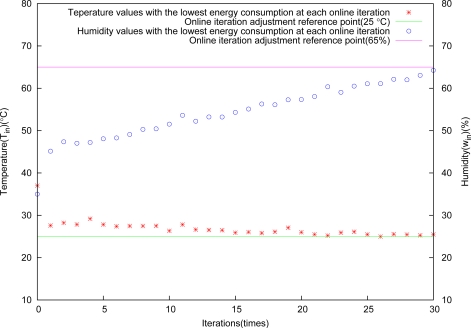
Control results through arithmetic sequence adjustment.

The control result is shown in Figure 8. It is obvious from this figure that the algorithm performs very well for greenhouse environment control.

**Figure 9.**
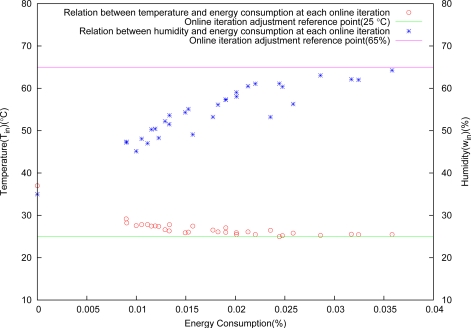
Relation between energy consumption and control results.

Temperature and humidity versus iterations almost appear linear, and they are in good agreement with the arithmetic sequence adjustment for the control deviation tolerance Δ*x* at each iteration. Figure 9 shows the relation between objective control points and energy consumption, and the higher control precision, the more energy consumption, because they are conflicting with each other.

### 6.2. Case 2: Fast Adjustment

To achieve faster control response speed, suppose the control deviation tolerance Δ*x* is modified as follows:

$$\begin{array}{c} \Delta x_{i+1}=\min(\Delta x_{i},\;\Delta {\mathrm{err}}_{i})\\ i=0,1,2,\ldots ,\mathrm{iter}\_\max-1. \end{array}$$

where Δ*err_i_* is the state variables deviation of the *i*th iteration (*i.e.*, Δ*err_i_* = |*x_i_* − *r*|).

**Figure 10.**
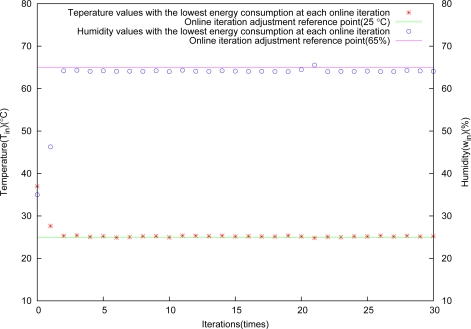
Control results through fast adjustment.

The fast control effect is shown in Figure 10. Temperature and humidity are nearly close to reference points at the second iteration simultaneously. The state curves controlled approach to reference points, and it is very stable.

### 6.3. Case 3: Square Root Adjustment

Besides, suppose the control deviation tolerance Δ*x* is given as follows:

$$\begin{array}{c} \Delta x_{i+1}=\frac{\Delta x_{0}}{\sqrt{i+1}}\\ i=0,1,2,\ldots ,\mathrm{iter}\_\max. \end{array}$$

**Figure 11.**
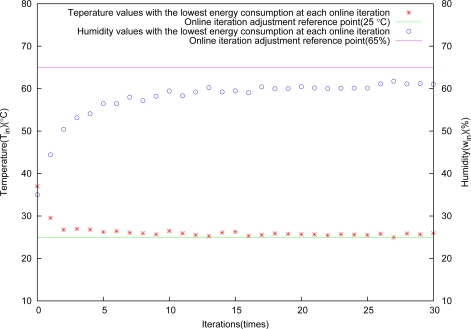
Control results through square root adjustment.

The result is shown in Figure 11. Temperature is quickly controlled to enter into the desired compatible objective regions at the second iteration. In contrast, humidity is slowly approaching to the control region with increasing iteration.

### 6.4. Case 4: Robust Control

Robust control seeks controllers that provide robust stability and performance for uncertain plants. In order to validate the robustness of the controller, we adopt the same adjustment method with case 3 and compare different control effects under the condition of various external disturbances. Suppose a group of external disturbances are constant given as follows:

$$v=\left(\begin{array}{c} v_{1}\\ v_{2}\\ v_{3} \end{array}\right)=\left(\begin{array}{c} T_{\mathrm{out}}\\ w_{\mathrm{out}}\\ S_{i} \end{array}\right)=\left(\begin{array}{c} 2\\ 25\\ 5 \end{array}\right)$$

and another group are given as follows:

$$v=\left(\begin{array}{c} v_{1}\\ v_{2}\\ v_{3} \end{array}\right)=\left(\begin{array}{c} T_{\mathrm{out}}\\ w_{\mathrm{out}}\\ S_{i} \end{array}\right)=\left(\begin{array}{c} -2+8*\mathrm{randf}()\\ 25+25*\mathrm{randf}()\\ 5+15*\mathrm{randf}() \end{array}\right)$$

**Figure 12.**
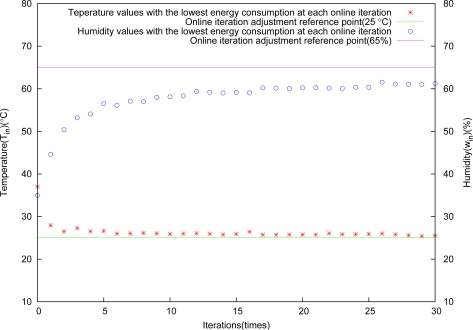
Robust comparative results by exerting constant disturbances (square root adjustment).

**Figure 13.**
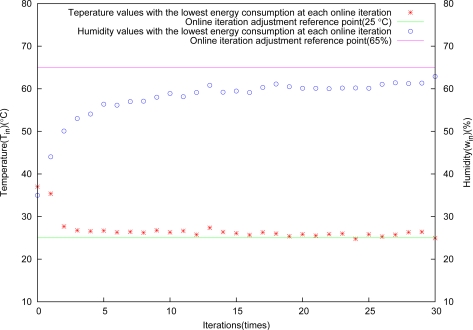
Robust comparative results by exerting random disturbances (square root adjustment).

The results by exerting the above external disturbance on the greenhouse environment are shown in Figure 12 and Figure 13, respectively. Compared with each other, Figures 11–13 show that control trajectories are nearly similar, *i.e.*, the controller is insensitive to changes in the greenhouse environment and can maintain their stability and performance under the uncertain environment conditions. The experimental results show that the proposed control method has a good robustness to the change of external climate condition.

### 6.5. Case 5: Open-Loop Control

**Figure 14.**
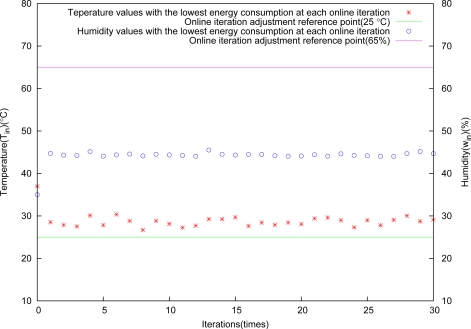
Open-loop control results.

Finally, we adopt an open-loop control architecture to validate the performance. The obtained population at each iteration is directly involved in the next iterative optimization, and the outputs have no influence on the inputs. It is shown in Figure 14 that temperature and relative humidity controlled fluctuate at 29 °C and 45%, respectively. Neither of them is controlled to the desired compatible objective regions. Especially, the relative humidity controlled is only 45%, not suitable for plant growth. In addition, the fluctuation range of temperature is drastic compared with relative humidity. Compared with the above close-loop control architecture, it is obvious that the stability and robustness of open-loop control are worse than close-loop control.

According to the above discussions, it is clearly shown that the proposed compatible control algorithm is an effective control algorithm. It has a very good stability and robustness for greenhouse environment control. To achieve as low energy load as possible, we have a preference selection for the solutions with the lowest energy consumption at each iteration, so as to get the minimum opening of equipment operation of the greenhouse for short time-scale according to the above optimal results. In order to control longer time periods, the control is divided into a 15 minute piece and the end states of one simulation are used as start values of the next period. By this means, we can set a favorable climate environment for the crop and achieve high yield at low expense, good quality and low environmental load.

Compared with our previous practical application of control methods based on experience and fuzzy prediction in the greenhouse, although the previous methods are simple and easy to implement, the compatible optimal control methods have many advantages. For example, when using experience-based methods, we always take measures to make up the control defects after the damages for plants happened, which usually causes the loss and high costs. In contrast, when using the compatible control methods, we select control parameters to implement objectives control according to the optimal solutions ahead, so we can effectively avoid the former disadvantages and can achieve lower energy consumption.

It should be noted that this study has examined only a simple greenhouse model. We only concentrated on the part disturbances and control components such as solar radiation, temperature and air humidity. In addition, the algorithm is time-consuming, but in practice it is enough for greenhouse climate control because the greenhouse itself is a large time delay system. Not withstanding its limitation, this study does suggest the use of the proposed approach to more complex and real-world engineering optimal control problems in greenhouse environment. These limitations could be solved if we consider more accurate and complex greenhouse dynamic model.

## 7. Conclusions

Conventional methods used for solving greenhouse environment multi-objective conflict control problems always select the optimal value of each performance as the point control objective and minimize the deviation of each performance. The resulting solution, therefore, causes high energy cost. Moreover, it is very difficult to achieve simultaneously the multiple objectives’ precise points control owing to the conflicts between the objectives.

In this paper, we proposed a modified multi-objective compatible control algorithm by reformulating the point control objectives into the interval or region control objectives, namely enlarging the point objectives to intervals or regions to ensure energy saving and the existence of the control solutions. The algorithm adopts two hierarchical levels, referred as the compatible optimization level and compatible control level. In the former, we can achieve the Pareto optimal fronts of control variables (*i.e.*, heating, fogging and ventilation) by calling the multi-objective optimizer according to the special requirements for plant growth and user’s experiences of greenhouse production. In the latter, based on the feedback control architecture, the indoor temperature (*T_in_*) and humidity (*w_in_*) can be regulated within the compatible objective regions by adjusting the control deviation tolerance.

The effectiveness of the proposed approach has been validated through various comparative studies with various adjustments for the control deviation tolerance Δ*x* and the open-loop regulator existing on the site based on a simple greenhouse heating/cooling/ventilating model. The results are encouraging and suggest the energy-saving application to more complex and real-world engineering problems in greenhouse production. It may be valuable and helpful to formulate environmental control strategies to pursue energy saving and to gradually realize the ultimate objective of environmental optimal control. Moreover, the proposed approach has potential to be useful for other practical control optimization problems with two characteristics: (1) there exist multiple competitive control optimization objectives; (2) these objectives can be allowed to settle for suboptimal solutions owing to the trade-off between multiple competitive specifications. The control of urban traffic flow is such a problem.

A first extension of the approach could be to find a more advanced controller for greenhouse environment control. In future research we will consider the design for the controller by employing multi-objective conflict compatible control algorithms.

## Figures and Tables

**Table 1.** Operators and parameters of compatible optimization.

Description	values
Population size	400
Number of generations	200
Probability of crossover of real variable	0.9
Probability of mutation of real variable	0.5
Distribution index for crossover	10
Distribution index for mutation	20

**Table 2.** Identified greenhouse model parameters.

Parameters name	unit expression	values
*C*_0_	*minW* °C^−1^	−324.67
*UA*	*W* °C^−1^	29.81
*t_v_*	*min*	3.41
λ′	*W*	465
α′	*gm*^−3^*min*^−1^*W*^−1^	0.0033
1/*V*′	*gm*^−3^*min*^−1^	13.3

**Table 3.** Operators and parameters of greenhouse climate.

Operators and parameters	values
Indoor initial temperature (°C)	37
Indoor initial humidity (%)	35
weight *ω*_1_	0.75
weight *ω*_2_	0.2
weight *ω*_3_	0.05
Initial temperature compatible objective regions (°C)	[15,35]
Initial humidity compatible objective regions (%)	[45,85]
Max iteration *iter_max*(times)	30
Control step (minutes)	15
